# In Vitro Mimicking of Obesity-Induced Biochemical Environment to Study Obesity Impacts on Cells and Tissues

**DOI:** 10.3390/diseases10040076

**Published:** 2022-10-03

**Authors:** Abdelaziz Ghanemi, Mayumi Yoshioka, Jonny St-Amand

**Affiliations:** 1 Department of Molecular Medicine, Faculty of Medicine, Laval University, Quebec, QC G1V 0A6, Canada; 2 Functional Genomics Laboratory, Endocrinology and Nephrology Axis, CHU de Québec-Université Laval Research Center, Quebec, QC G1V 4G2, Canada

**Keywords:** obesity, environment, cell, tissue, mechanisms

## Abstract

Obesity represents a heavy burden for modern healthcare. The main challenge facing obesity research progress is the unknown underlying pathways, which limits our understanding of the pathogenesis and developing therapies. Obesity induces specific biochemical environments that impact the different cells and tissues. In this piece of writing, we suggest mimicking obesity-induced in vivo biochemical environments including pH, lipids, hormones, cytokines, and glucose within an in vitro environment. The concept is to reproduce such biochemical environments and use them to treat the tissue cultures, explant cultures, and cell cultures of different biological organs. This will allow us to clarify how the obesity-induced biochemistry impacts such biological entities. It would also be important to try different environments, in terms of the compositions and concentrations of the constitutive elements, in order to establish links between the effects (impaired regeneration, cellular inflammation, etc.) and the factors constituting the environment (hormones, cytokines, etc.) as well as to reveal dose-dependent effects. We believe that such approaches will allow us to elucidate obesity mechanisms, optimize animal models, and develop therapies as well as novel tissue engineering applications.

## 1. Modern Health Challenges and Obesity

Modern life is characterized by diseases and health problems, some of which are emerging, and others that already existed but have worsened. We see the emergence of new pathologies such as viral and bacterial infections with new microbiological agents or new variants of those agents. On the other hand, another category of previously existing diseases is worsening. These diseases include obesity, cancer, and cardiovascular diseases. Such an epidemiological profile is mainly due to the unhealthy lifestyle developed by our societies during the last decades. This poor lifestyle includes the lack of physical activity [1] resulting from sedentary habits, as well as the technological and scientific developments that have made it easier to complete our daily tasks with limited or no effort. Psychological and mental health issues are also among the factors that worsen our lifestyle. Importantly, the contribution of dietary choices—in terms of both quality and quantity—to lifestyle quality is tremendous [2]. Within this context, we focus on obesity and the related biochemical environment it induces in vivo to build in vitro models of the disease.

Obesity, as a health problem [3,4] that has reached pandemic levels [5], is a disease with complex pathogenesis and complications [6]. Its basic definition is an abnormal fat accumulation as a consequence of energy imbalances between caloric intake and energy expenditure [7,8]. Obesity is characterized by adipocytes proliferation [8,9] and adipose tissue growth and remodeling [10,11,12]. Obesity properties have even been compared to cancer [13], and the disease has also been described as neuroendocrine reprogramming [14]. 

Beyond the impacts that obesity has via biomechanical mechanisms, such as on joints (cartilage loss) [15] and lungs (mechanics changes) [16], obesity has also been suggested either as a cause or a risk factor for impaired regeneration [17], arterial hypertension [18,19], type 2 diabetes mellitus [20,21], nonalcoholic fatty liver disease [22,23], heart failure [24,25], kidney diseases [26,27], cancer [28,29,30], dyslipidemia [31,32], etc. Functional genomics explorations of obesity and the factors that impact its development (diet, exercise, etc.) [33,34,35,36,37,38,39,40,41] have revealed specific related genes and improved our molecular understanding of obesity development. 

It is worth mentioning that diet not only contributes to obesity development via increased energy intake, but also via its indirect effects [42]. Indeed, various food elements (vitamins, antioxidants, etc.) can impact the performance of the metabolic functions and biochemical homeostasis. In addition, a diet that results in obesity also contributes to the metabolic consequences and biological environments (blood contents in lipids, glucose, etc.) developed once obesity and the obesity-related homeostasis impairments are established.

## 2. In Vitro Obesity-Related Environment 

The impacts that obesity-related chemical and biological environments have on homeostasis, as well as on the cellular and tissular functions, along with the underlying pathways, remain less known. Importantly, understanding the homeostatic patterns related to obesity-induced biochemistry and molecular changes would help to better explain how obesity leads to or increases the risks of the pathological phenotypes seen in diverse tissues and organs. Indeed, obesity status is also characterized by a biological environment created from the changes in secretion and biomolecules levels changes seen in the disease. These include dyslipidemia [43]; impaired acid-base balance, as reflected in the urine pH and the small intestine pH [44,45,46,47,48,49]; chronic inflammation [50,51], including gut microbial-related low-grade inflammation [52]; pro-inflammatory cytokines production [53,54,55]; hyperinsulinemia [56,57,58]; thyroid hormones and gender steroids (among other endocrine changes) [59]; increased leptin [60,61]; metabolic dysfunction [62,63]; decreased adipose tissue oxygenation [64]; and hyperuricemia [65,66,67,68]. 

Such biochemical and molecular changes reported in obesity could represent an important component of the mechanistic links between obesity and its consequences on cells and tissues. Therefore, building in vitro models to explore such mechanistic pathways would allow us to develop studies that are geared towards a deeper understanding of the obesity pathogenesis, development, and the impacts that it has on the diverse cells, tissues, and organs. The purpose is to investigate and explore the changes that such obesity-induced biochemical environments induce on cells and tissues in vitro and explore the modified mechanisms based on the observed in vitro changes.

**Figure 1:**
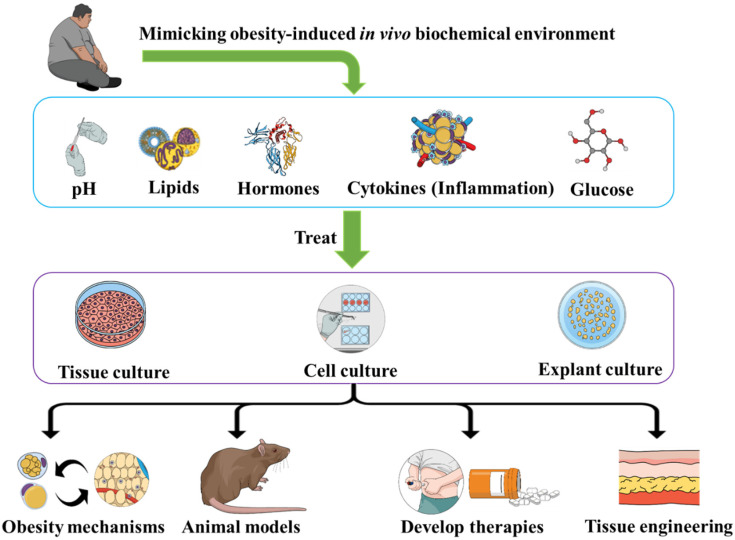
Treating cells and tissues with an obesity-like bioenvironment allows us to study obesity, develop animal models and therapies, as well as optimize tissue engineering methods.

The concept is to place different types of cell [69] or tissue cultures within environments (culture medium) that mimic the in vivo obesity environments in terms of pH [70,71], inflammatory factors, insulin [72,73], hormonal factors [74,75], lipids content, glucose, etc. (Figure 1). Testing different types of cells/tissues cultures would allow us to build a database towards a molecular mapping of the effects of each set of conditions (one or more factors within the culture medium) on different types of cells/tissues (adipocytes, renal cells, hepatocytes, myocytes, muscles, etc.) and identify the cellular and molecular outcomes. Furthermore, to obtain “dose-dependent” patterns, we can use different compositions of the medium in terms of concentrations of diverse elements within the media. The factors to use (hormones, cytokines, biochemicals, etc.) could also be optimized according to the biological samples, especially depending on the receptors of these factors on the cells/tissues of the culture. The time of exposure should also be a variable of focus, in order to distinguish acute exposure outcomes and chronic effects (more relevant to obesity). 

Such approaches would allow us to better understand how obesity-related biochemistry induces modifications within the biology of the cells and tissues. By studying different media compositions, including media in which we add only one parameter (for instance cytokines such as interleukin-1 beta (IL)-1β and IL-6 and tumor necrosis factor-alpha (TNF-α) [53]), we could be able to link each effect of obesity to one or more factors. Therefore, we could develop therapies to manage obesity consequences depending on the symptoms and target the observed phenotype, especially with the importance of cell culture in drug development [76,77,78]. Such therapy would be more specific since we would know which part of the obesity-induced biochemistry (hormone, pH, etc.) is responsible for the pathological phenotype (kidney, liver, etc.). Interestingly, applying such approaches on embryonic and stems cells could help in understanding the effects of obesity-related biochemistry on embryos (whose mothers develop obesity since they share the blood circulation for which the biochemistry is modified by obesity), and on the biology of development in general. The other key point is that such in vitro methodologies (mainly cell cultures, explant cultures, and tissue cultures) would allow us to study and observe a specific obesity-induced cellular phenotype independently from the other factors, and thus, specifically understand its pathogenesis and the influencing factors. As practical illustrations, recent research papers applied similar concepts to generate fat-on-a-chip models [79], an in vitro model for hypertrophic adipocytes [80], and inflammation in an in-vitro model of central obesity [81], all for applications in obesity discovery and research. These examples are practical illustrations of in vitro models of obesity that would provide strong data to support, confirm, and complete the data collected from animal models. These in vitro models can also allow us to overcome certain limitations reported in animal models by allowing us to study the interactions between one type of cell, tissue, or organ with one factor or one set of factors (signalizing molecules, cytokines, etc.), independently from the other factors. This is not always possible in the animal models since an in vivo environment includes all the factors that are also in variable concentrations with continuous dynamic changes.

## 3. Biomedical and Clinical Perspectives

Biomedical and clinical perspectives would add important knowledge to the diverse animal models of obesity [82,83,84,85,86], since precise knowledge on the changes that obesity induces in vivo can be applied to further optimize these animal models. This can be achieved, for instance, by injecting chemicals to maximize the biochemical similarities between the patients suffering from obesity and the in vivo patterns of the obesity animal models. In addition, a good example of clinical application would be in regenerative medicine. Indeed, understanding the links between the obesity-induced environment and tissues development could allow us to optimize the tissue culture conditions towards more regenerative medicine-produced tissues for therapies, especially for patients with obesity. This is specifically important due to the specific factors (such as AMP-activated protein kinase and mitochondrial biogenesis) and the related effects of impaired regeneration in the context of obesity [87,88,89,90].

The above-explained principle can be extrapolated to other diseases/conditions that could induce biochemical changes impacting cells, organs, and tissues, such as diabetes, and ageing that shares patterns with obesity [91,92]. Further, it would allow us to explain how the biochemical changes induced by these health conditions could mechanistically lead to the known phenotypes in the diverse tissues (metabolic decline or disorder, cellular senescence, etc.). As illustrated, exposing cell cultures to oxidative stress would allow us to deeply explore the theory of ageing related to free radicals [93,94].

However, it is important to mention the limitations, because the in vitro investigation of cell, tissue, or explant cultures may not completely mimic the in vivo obesity environment, and differences between in vitro and in vivo environments exist. For instance, the surrounding environments’ constitutive elements (hormones, cytokines, etc.) have fixed concentrations in the in vitro cultures, whereas in vivo, their production and concentration changes and are under the control of different regulating factors. Unlike the in vitro conditions, the in vivo mechanisms ensure that both signaling molecules (hormones, cytokines, etc.), as well as the biometabolites (fatty acids, glucose, etc.), have variable patterns of production and secretion depending on food intake, controlling signals, and feedback control pathway among a regulating biological network.

We hope that the knowledge we gain through these approaches will provide additional tools to manage obesity in an era where this disease [95,96] impacts continue to worsen, as illustrated by the COVID-19 crisis [97,98,99,100], since gaining such molecular and genetic knowledge about obesity is the main obstacle towards a deep understanding of obesity-related pathways and, therefore, developing efficient therapies. Building this kind of database would allow us to develop new treatments or improve those already in use. Moreover, the biochemicals that comprise the obesity environment could also be a target of the therapies (develop pharmacotherapies) or be modified by anti-obesity approaches such as exercise [101], which could allow a better understanding of how exercise improves obesity beyond caloric balance and weight loss. This could not only explain obesity pathogenesis, but also provide mechanistic explanations of how anti-obesity therapies work. Importantly, the significance and implications of the present piece of writing in clinical applications and therapies is related to the fact that obesity is a growing problem worldwide and is associated with a range of health complications, including diabetes and hyperlipidemia. Moreover, these kinds of in vitro models represent suitable models for pharmacodynamics-related studies aiming to explore the changes of drug–target interactions, depending on whether the surrounding medium mimics the obesity-induced bioenvironment. Therefore, we could provide data towards optimized therapies for patients with obesity, as well as potentially improve the pharmacovigilance of obesity therapies.

## Data Availability

Not applicable.
